# Supplementary material for: A Nano-Emulsion Platform Functionalized with a Fully Human scFv-Fc Antibody for Atheroma Targeting: Towards a Theranostic Approach to Atherosclerosis
Source: Int J Mol Sci. 2021 May 14;22(10):5188. doi: 10.3390/ijms22105188 (PMC8153629; doi:10.3390/ijms22105188)
Supplement: Supplementary file 1 [file ijms-22-05188-s001.zip › Supporting Information_IJMS.docx]

*Article*

# Supplementary Information

**A Nano-Emulsion Platform Functionalized with a Fully Human scFv-Fc Antibody for Atheroma Targeting: Towards a Theranostic Approach to Atherosclerosis**

Samuel Bonnet^1,2,^*^,†^, Geoffrey Prévot ^3,†^, Stéphane Mornet ^2^, Marie-Josée Jacobin-Valat ^1^, Yannick Mousli ^3^,
Audrey Hemadou ^1^, Mathieu Duttine ^2^, Aurélien Trotier ^1^, Stéphane Sanchez ^1^, Martine Duonor-Cérutti ^4^,
Sylvie Crauste-Manciet ^3,†^ and Gisèle Clofent-Sanchez ^1,^^†^

^1^ CNRS UMR 5536, Université de Bordeaux, CRMSB, Centre de Résonance Magnétique des Systèmes
Biologiques, 33076, Bordeaux, France; samuel.bonnet@univ-angers.fr (S.B.); marie-josee.jacobin-valat@rmsb.u-bordeaux.fr (M.-J.J.-V.); audrey.hemadou@orange.fr (A.H.); aurelien.trotier@rmsb.u-bordeaux.fr (A.T.); stephane.sanchez@rmsb.u-bordeaux.fr (S.S.); gisele.clofent-sanchez@rmsb.u-bordeaux.fr (G.C.-S.)

^2^ CNRS UMR 5026, Université de Bordeaux, Bordeaux INP, ICMCB, Institut de Chimie de la Matière
Condensée, F-33600, Pessac, France; samuel.bonnet@univ-angers.fr (S.B.); Stephane.Mornet@icmcb.cnrs.fr (S.M.); Mathieu.Duttine@icmcb.cnrs.fr (M.D.);

^3^ Université de Bordeaux, INSERM U1212, CNRS UMR 5320, ARNA, ARN, Régulations Naturelle et Artificielle, ChemBioPharm, 33076, Bordeaux, France; geoffrey.prevot@gmail.com (G.P.); ymousli33@gmail.com (Y.M.); sylvie.crauste-manciet@u-bordeaux.fr (S.C.-M.)

^4^ CNRS UPS 3044, Baculovirus et Thérapie, 30380, Saint-Christol-lès-Alès, France; Martine.CERUTTI@cnrs.fr (M.D.-C.)

***** Correspondence: samuel.bonnet@univ-angers.fr

† These authors contributed equally.


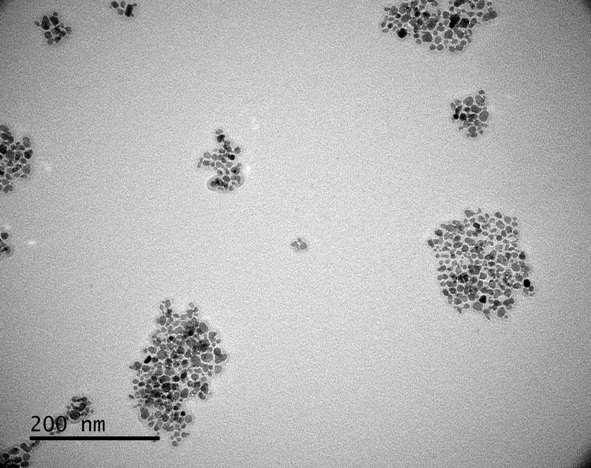


**Figure S1.** Transmission electron microscopy (TEM) picture without negative staining of the nanoemulsions loaded with magnetic particles

**Video S1.** Example of dynamic correction

Video.mp4
