# Supplementary figures and images for: A Nano-Emulsion Platform Functionalized with a Fully Human scFv-Fc Antibody for Atheroma Targeting: Towards a Theranostic Approach to Atherosclerosis
Source: Int J Mol Sci. 2021 May 14;22(10):5188. doi: 10.3390/ijms22105188 (PMC8153629; doi:10.3390/ijms22105188)

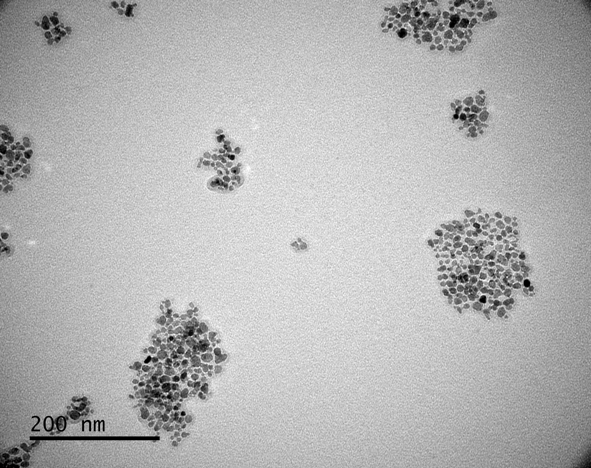

Supplement: Supplementary file 1 [file ijms-22-05188-s001.zip › TEM.png]
